# Conservative Vertical Groove Technique for Tooth Rehabilitation: 3-Year Follow-Up

**DOI:** 10.1155/2018/2012578

**Published:** 2018-01-28

**Authors:** Dhanalaxmi Karre, Mahesh Kumar duddu, Silla Swarna Swathi, Abdul Habeeb Bin Mohsin, Bhogavaram Bharadwaj, Sheraz Barshaik

**Affiliations:** ^1^Department of Pedodontics, Sri Sai College of Dental Surgery, Vikarabad, India; ^2^Department of Pedodontics, G Pulla Reddy Dental College, Kurnool, India; ^3^Department of Prosthodontics, Sri Sai College of Dental Surgery, Vikarabad, India; ^4^Department of Oral and Maxillofacial Surgery, Sri Sai College of Dental Surgery, Vikarabad, India; ^5^Department of Oral and Maxillofacial Surgery, MNR Dental College & Hospital, Sangareddy, India

## Abstract

Reattachment of tooth fragment is a simple, conservative, and noninvasive procedure, and it is the most currently acceptable treatment option. This article presents management of two accidentally damaged maxillary incisors using direct composite resin restoration and fractured tooth fragment. With the advancements in adhesive dentistry, tooth fragment reattachment procedure has become simpler and clinically reliable. The present paper is a report of 3-year follow-up of coronally fractured tooth treated with a very conservative technique of tooth fragment reattachment using vertical groove preparation and reinforcement with fiber post.

## 1. Introduction

Coronal fractures are the most common frequent form of traumatic dental injuries commonly involving maxillary incisors. This can be explained by their anterior placement and protrusion due to eruption pattern. Factors such as age, gender, race, and overjet predispose dental trauma in maxillary anterior teeth with higher prevalence in the age groups between 2-3 years and 8–12 years [1, 2]. Based on involvement of tooth structure, coronal fractures are broadly categorized as complicated and uncomplicated fractures [3]. Reconstruction of coronal fractures immediately is important for the positive psychological status and to maintain esthetics [4]. Although direct composite restoration has expanded over the past decade, fragment reattachment is increasingly opted due to its minimal invasion, promising esthetics, and a natural form of restoration [5]. In the present case, there was Ellis class II fracture of maxillary left central and lateral incisors. Tooth fragment for maxillary left central incisor was not available; hence, it was reconstructed with direct composite restoration. Maxillary left lateral incisor was managed with fragment reattachment using vertical groove technique followed by fiber post placement in the vertical groove thus offering higher esthetics and improved function.

## 2. Case Report

**Figure 1:**
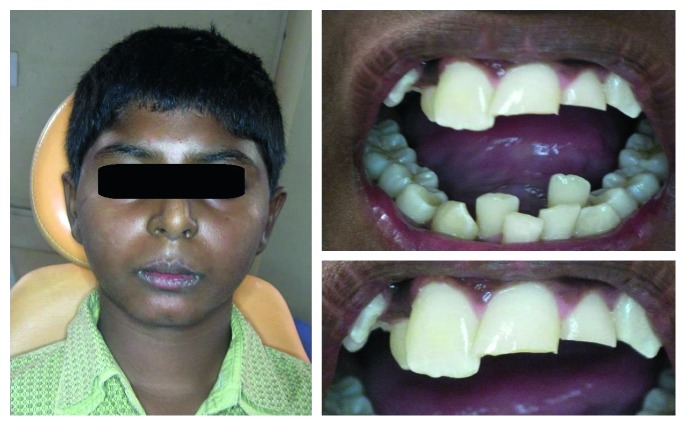
Preoperative view showing psychologically disturbed boy due to Ellis class II fractured teeth 21 and 22.

A 12-year-old boy reported to the Department of Pedodontics with fractured maxillary incisors due to sudden strike on the wooden bench. There was no history of loss of consciousness and vomiting. His parent brought intact tooth fragment in a water filled container. Clinical examination revealed fracture of maxillary left central and lateral incisors involving enamel and dentin with no pulp exposure (Figure 1). There was a lacerated labial gingiva in the mandibular incisors region. Periapical radiograph of maxillary left central and lateral incisors showed absence of alveolar fracture with intact roots and closed apices with no periapical pathology. Considering various treatment options available, reattachment of tooth fragment of maxillary left lateral incisor and direct composite restoration of maxillary left central incisor was planned. The chosen treatment was a less invasive technique which promotes an immediate repair of the esthetics and function of fractured tooth. Treatment was explained to parents, and an informed consent was taken.

**Figure 2:**
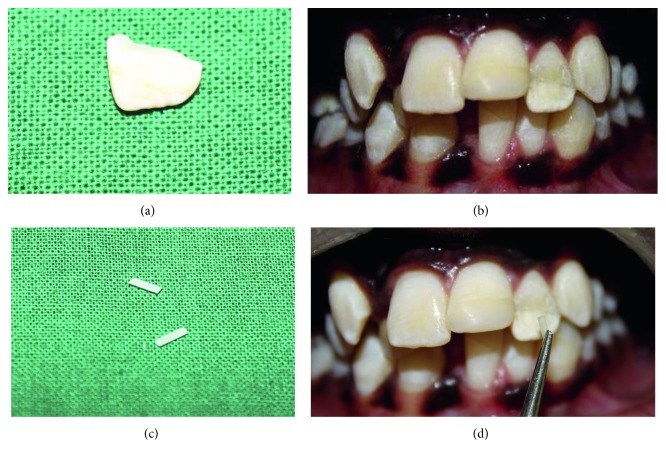
Intact tooth fragment of tooth 22 (a). Composite reconstruction of tooth 21 and fragment reattachment with vertical groove placement of tooth 22 (b). Fiber post sectioned and placed in prepared vertical grooves (c, d).

**Figure 3:**
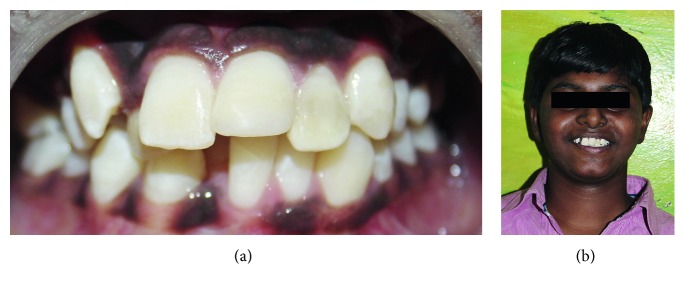
Immediate postoperative picture (a). 3-year follow-up picture showing intact tooth fragment (b).

Under strict asepsis condition, bilateral mental nerve block was administered using 2 percent lidocaine with 1 : 80000 adrenaline, and lacerated labial gingiva was sutured. Composite reconstruction of maxillary left central incisor was performed with total etch multilayered technique. The fractured component and tooth structure of maxillary left lateral incisor were cleaned and acid etched with 37% orthophosphoric acid gel for 20 seconds. After thorough rinsing and drying, adhesive was placed on both tooth fragment (Figure 2(a)) and tooth structure and air thinned and light cured for 10 seconds. Tooth fragment was reattached using low-viscosity flowable resin cement and light cured. After reattachment, two vertical grooves of depth and width 2 mm were placed along the fracture line on the labial surface using depth orientation bur (Figure 2(b)). Fiber-reinforced composite posts (quartz no. 1) were cut of the same size and placed in the prepared grooves (Figure 2(c) and 2(d)). Posts were attached to tooth using resin cement and light cured. Excess composite material was removed, finished, and polished (Figure 3(a)). The patient was advised dietary, oral hygiene instructions and recalled after one week for the suture removal. One-year follow-up showed successfully retained fragment of maxillary left lateral incisor while composite restoration of maxillary left central incisor was dislodged; hence, reconstruction of dislodged composite restoration was done again. 2-year and 3-year follow-up showed successfully retained fragment and composite restoration serving their function, and the patient was asymptomatic throughout the period (Figure 3(b)).

## 3. Discussion

Preservation of healthy tissue, longevity, esthetics, and function of restored tooth structure represented major objective of restorative dentistry [6]. Traumatized teeth with coronal fractures can be managed by various techniques such as resin crowns, ceramic crowns, orthodontic bands, and composite restoration with or without posts [7]. With the development of adhesive dentistry, reattachment of tooth fragment is considered as the best alternative for restoring coronal fractures in anterior teeth. Tooth fragment reattachment is far superior to direct composite restoration as enamel is maintained thus restoring natural color, contour of the fractured tooth, and it is considered as a viable treatment alternative. Conceição reported fragment reattachment as a simple, safer, and extremely conservative technique offering excellent esthetics and maintaining occlusal function [8]. Crucial aspects in the fragment reattachment technique are the location of the fracture, adaptation of the fragment to the remaining tooth structure, size of the fragment, and its hydration [9, 10]. In the present case, availability of tooth fragment with satisfactory surface area and size indicated fragment reattachment technique for maxillary left lateral incisor.

Various factors play a vital role in longevity and function of reattached fragment. These factors include storage media used for tooth fragment, material, and technique used for fragment reattachment. According to the recent literature, there is no effect on fracture strength when a different resin material was used. Many techniques are proposed for fragment reattachment which include simple reattachment, chamfering, over contour, and internal dentin groove. In simple reattachment technique, only bonding is done without any additional wearing of tooth fragment or remaining tooth structure [11, 12]. External chamfer, over contour, and internal dentin groove techniques help in obtaining adequate function, retention, and esthetics.

In this case, vertical groove technique was used in which two vertical grooves were prepared along the fracture line creating space for fiber post placement thus increasing the fracture strength of retained tooth fragment. Low-viscosity resin was used for reattachment of tooth fragment to obtain minimum thickness along cementation line. Fiber post was used extracoronally as it was an uncomplicated crown fracture. Studies have reported that fragment reattachment is superior to direct composite restoration as higher fracture strength can be attained [13]. In the present case, over 1-year follow-up, there was dislodged composite restoration while tooth restored with fragment reattachment was intact and functional. Fragment reattachment should be attempted in priority in young children as it showed higher retention rate and better esthetics and boosts psychological confidence when compared to other treatment options [14].

## 4. Conclusion

Fragment reattachment is an esthetically acceptable and a conservative approach in the management of traumatic dental injuries. This procedure of vertical groove preparation and fiber post showed excellent stabilization even after a 3-year follow-up, and it was esthetically pleasing with no color change.
